# LncRNA C2orf27A Promotes Gastric Cancer by Sponging MiR-610 and Elevating NOX4 Expression

**DOI:** 10.7150/jca.100621

**Published:** 2025-01-27

**Authors:** Mingkai Zhuang, Xiaoxiong Guo, Dan Lin, Na Lin, Xiaozhong Wang, Fenglin Chen

**Affiliations:** 1 Department of Gastroenterology and Fujian Institute of Digestive Disease, Fujian Medical University Union Hospital, Fuzhou, Fujian 350001, China;; 2 Fujian Medical University Cancer Center, Fujian Medical University, Fujian 350001, China.; 3 Fujian Clinical Research Center for Digestive System Tumors and Upper Gastrointestinal Diseases, Fujian Medical University, Fuzhou, Fujian 350001, China.

**Keywords:** LncRNA C2orf27A, MiR-610, NOX4, CeRNA, Gastric Cancer

## Abstract

Long non-coding RNAs (lncRNAs) are crucial for gastric cancer (GC) progression. In this study, we aimed to investigate the function and molecular pathways of lncRNA C2orf27A in GC development. Bioinformatics databases, tissue cDNA microarrays, and cell lines were used to assess the expression of C2orf27A in GC. Cell proliferation was assessed using Cell Counting Kit-8, colony formation, cell cycle assays, whereas cell death using the Annexin V-APC/7-AAD assay. Subcutaneous xenograft mouse models were used to assess the effects of the C2orf27A knockdown on GC growth *in vivo*. The subcellular localization of C2orf27A in GC cells was verified using nucleocytoplasmic separation. Bioinformatics analysis predicted the binding of C2orf27A, miR-610, and NADPH oxidase 4 (NOX4), which was validated using dual luciferase reporter gene assay. We found that C2orf27A expression increased in GC tissues and cells. Furthermore, GC patients with increased C2orf27A expression levels had worse survival rates. Silencing of C2orf27A suppressed GC cell growth and induced GC cell death *in vitro* and *in vivo*. Further investigations into underlying mechanisms showed that C2orf27A functions as a competitive endogenous RNA against miR-610, leading to increased NOX4 expression levels in GC cells. Notably, blocking miR-610 and increasing NOX4 expression levels reversed the anticancer effects of reduced C2orf27A levels in GC cells. In summary, C2orf27A promotes cell proliferation and reduces cell death through the miR-610/NOX4 pathway in GC, which may provide a new perspective for further elucidation of the molecular mechanism underlying GC progression.

## Introduction

Gastric cancer (GC) is a prevalent malignancy, with approximately 1 million new cases and 770,000 deaths annually 1. Despite advances in surgery, chemotherapy, and radiotherapy, the long-term survival of GC remains low because of the lack of adequate early diagnosis and appropriate molecular biomarkers. Therefore, the development of new effective biomarkers and therapeutic targets is necessary to improve the prognosis of GC patients.

Long non-coding RNAs (lncRNAs) are RNA molecules that are longer than 200 nucleotides and do not appear to encode proteins. They are associated with various aspects of cancer occurrence and development. Recent studies have shown that several lncRNAs are abnormally expressed in different cancer types and regulate the aggressiveness of cancer cells, indicating their potential as biomarkers for cancer detection and prediction 2. Furthermore, accumulating evidence indicates that lncRNAs are significantly involved in GC progression, impacting cell growth, death, movement, invasion and resistance to chemotherapy and radiotherapy 3, 4.

The lncRNA C2orf27A is located on chromosome 2q21.1-q21.2 and spans 2308 base pairs. It was discovered in 2021 as a crucial gene in hepatocellular carcinoma (HCC) linked to sorafenib resistance and acts as a standalone prognostic indicator of cancer stages and tumor grades 5. Moreover, a previous study used computational analysis to predict that C2orf27A could potentially affect genes associated with ferroptosis and hinder the activation of CD4+ T cells, resulting in negative outcomes in patients with GC 6. Nonetheless, further experimental evidence is necessary to elucidate the functional roles and underlying mechanisms of C2orf27A in GC.

LncRNAs modulate gene expression through interactions with microRNAs (miRNAs), which typically inhibit gene expression 7. For example, miR-610 inhibits cancer and its expression is significantly downregulated in various malignant tumors 8-10. Moreover miR-610 hinders the movement and infiltration of GC cells by suppressing the activity of vasodilator-stimulated phosphoprotein 11. NADPH oxidase 4 (NOX4), a member of the NOX family, is central to the production of reactive oxygen species (ROS), which play a role in tumor cell proliferation, apoptosis, and various biological functions 12. Recent studies have indicated that NOX4 is overexpressed in GC and positively associated with tumor size and prognosis. Furthermore, studies on molecular processes have shown that the NOX4-triggered ROS production controls the growth and death of GC cells via the GLI1 pathway 13. Previously, our bioinformatics analysis demonstrated the existence of complementary binding sites between C2orf27A and miR-610, as well as between miR-610 and NOX4 mRNA. Thus, our findings suggested that C2orf27A might promote GC by interacting with miR-610 and NOX4.

In this study, we found that C2orf27A was highly expressed in GC tissues and correlated with the poor prognosis in patients with GC. C2orf27A knockdown significantly inhibited GC cells proliferation and promoted apoptosis *in vitro* and *in vivo*. Further investigations revealed that C2orf27A controlled the progression of GC by sponging miR-610 and functioning as a competitive endogenous RNA (ceRNA) of NOX4.

## Materials and Methods

### Bioinformatics analysis

The STAD dataset, containing 375 GC and 32 normal gastric tissue samples, was acquired from The Cancer Genome Atlas Program (TCGA, https://tcga-data.nci.nih.gov/tcga/). Differentially expressed lncRNAs were analyzed with the limma package in R software (version 3.6.3), using a threshold of |log2FoldChange| > 1 and an adjusted P-value < 0.05. An independent or a paired samples t-test was used to assess differences in C2orf27A expression levels between GC and normal gastric tissue samples. To enhance the standard sample size and bolster comparative analysis, we procured transcripts per million (TPM) data for typical human gastric tissue from GTEx (https://www.gtexportal.org/). Patients were allocated into a high- and low-expression group with TPM values above and below, respectively, the median TPM of C2orf27A expression. Subsequently, Kaplan-Meier survival analyses were conducted, and overall survival plots were created using the Survival and Survminer package based on TCGA-STAD datasets and the KMplot online database, which includes data of 875 patients with GC from six Gene Expression Omnibus (GEO) datasets (https://kmplot.com/analysis/index.php?p=service&cancer=gastric). The log-rank test was used to compare the two groups. The location of C2orf27A in cells was predicted using the lncLocator database (http://www.csbio.sjtu.edu.cn/bioinf/lncLocator/). The interaction between C2orf27A and miR-610 was predicted using the LncTar online tool, and the association between miR-610 and NOX4 was predicted using the DIANA-microT-CDS database (http://www.microrna.gr/microT-CDS).

### Cell culture

NCI-N87, HGC-27, and MKN-7 human GC cell lines and GES-1 normal human gastric epithelium cell line, were acquired from the Chinese Academy of Medical Sciences, and Wuhan Pricella Biotechnology Co. Ltd (China). The HEK-293 cell line, derived from the human embryonic kidneys, was obtained from Wuhan Pricella Biotechnology Co. Ltd. The HGC-27, MKN-7, and HEK-293 cell lines were cultured in Dulbecco's modified Eagle's medium (ProCell, China), whereas the NCI-N87 and GES-1 cell lines were cultured in RPMI-1640 medium (ProCell). The growth medium was supplemented with 10% fetal bovine serum and 1% dual antibiotics, specifically penicillin and streptomycin. All cells were cultured in a humidified environment with 5% CO_2_ at 37°C.

### Transfection of lentiviral vectors and oligonucleotides

Two short interfering RNA (shRNA) constructs targeting C2orf27A (shC2orf27A-1 and shC2orf27A-2) and a negative control shRNA (shNC) were designed, and their sequences were inserted into the pLVX-shRNA2-puro vector. The coding sequence (CDS) of human NOX4 was synthesized and cloned into the pLVX-IRES-ZsGreen1 vector. All inserted sequences were verified by DNA sequencing. The lentiviral particles were transfected into HEK-293 cells. A mimic of hsa-miR-610 was used instead of miR-610 and a chemically altered antisense oligonucleotide, an hsa-miR-610 inhibitor, was used to inhibit the expression of miR-610. The specific oligonucleotides used for knockdown and miRNA transfection are listed in Table S1. Cells, at a density of 1.2 × 10^6^ cells/well, were seeded in six-well plates to facilitate transfection. Once the cells reached 60-70% confluence, they were transfected with the corresponding lentivirus or oligonucleotides. Lipofectamine® 2000 reagent (Thermo Fisher Scientific, USA) was used to aid the transfection of oligonucleotides following the manufacturer's instructions.

### GC tissue cDNA microarray

A commercial source, Shanghai Outdo Biotech (China), supplied a GC tissue cDNA microarray (TMA). The TMA comprised 29 GC tissues and their corresponding adjacent normal gastric tissues. Adenocarcinoma was definitively diagnosed in all patients after surgery. The tissues were analyzed for C2orf27A expression levels using quantitative real-time PCR (qRT-PCR).

### Nuclear and cytoplasmic RNA fraction isolations

RNA was extracted from HGC-27 cells using a Cytoplasmic and Nuclear RNA Purification Kit (Norgen Biotek, Canada), in accordance with the manufacturer's instructions. Initially, a confluent 3.5 cm plate of cells was washed with cold PBS. Subsequently, 200 µL of cold lysis buffer was introduced into the culture plate, followed by gently tapping and swirling the plate for 5 min on ice. Then, the specimens were centrifuged at 14000 × g for 10 min at 4°C. The cytoplasmic fraction was separated from the nuclear fraction by using Buffer SK and Elution Buffer for RNA extraction. The RNA samples that had been purified were kept at -80°C until further analysis.

### RNA extraction and qRT-PCR

Total RNA was extracted from the cell lines using the TRIzol reagent (Thermo Fisher Scientific, USA). Reverse transcription was performed using the All-in-One™ First-Strand cDNA Synthesis Kit (GeneCopoeia, USA). The resulting cDNA, or a commercial tissue cDNA microarray, was subsequently used to examine the levels of C2orf27A, miR-610, NOX4, U6, and GAPDH using a qTower 3.2G Real-Time PCR System (Analytik Jena, Germany) and the BeyoFast™ SYBR Green qPCR Mix (Bio-Rad, USA). qRT-PCR was conducted with the following parameters for 40 cycles: initial denaturation at 95°C for 2 min, followed by denaturation at 95°C for 15 s, annealing and extension at 60°C for 30 s. GAPDH served as the internal control, with gene expression levels evaluated based on the threshold cycle value determined using the 2^-ΔΔCq^ method. Table S2 displays the primer sequences used for the genes studied are listed.

### Western blotting

Cells were lysed using RIPA lysis buffer (Beyotime, China), containing 1 mM phenylmethanesulfonyl fluoride. After centrifugation, the liquid above the sediment was collected and the amount of protein was measured using a BCA protein assay kit (Beyotime). Subsequently, 10% SDS-PAGE was used to separate 30µg of total cellular protein, which was subsequently transferred onto PVDF membranes from Millipore. After blocking with 10% skim milk for an hour at room temperature, the membranes were incubated overnight at 4°C with rabbit polyclonal antibodies against NOX4 (1:2000) and GAPDH (1:6000, Proteintech, China). After washing the membranes thrice with TBST buffer, they were incubated with secondary goat anti-rabbit antibodies for 1 h at room temperature. Protein bands were detected using enhanced chemiluminescence with a Tanon-5200 Multi Chemiluminescent Imaging System.

### Dual luciferase reporter gene assay

The binding sites of miR-610 in the C2orf27A or NOX4 sequences were predicted and mutated. These sequences were cloned into the psiCHECKTM-2 Vector (Promega, USA). Thereafter, miR-610 mimics or miR-610 inhibitor were co-transfected into HEK-293 cells with luciferase reporters using Lipofectamine® 2000 reagent. Relative luciferase activity was measured 48 h following the manufacturer's instructions using a Dual Luciferase Reporter Gene Assay Kit (Beyotime, China).

### Cell Counting Kit (CCK)-8 assay

Transfected cells were seeded in 96-well plates at a density of 5000 cells/well and incubated for 24, 48, and 72 h. Cell viability was assessed using a CCK-8 Kit (Sigma, USA) following the manufacturer's instructions. A microplate reader was used to measure the optical density at 450 nm.

### Colony formation assay

Typically, the processed cells were thinned out before being distributed into 200 cells and placed in six-well plates. These cells were then cultured in medium containing 10% FBS for 14 days. Subsequently, the colonies were treated with 4% formaldehyde for 10 min and stained with crystal violet for 5 min. Finally, the colonies were imaged and counted using the ImageJ software.

### Flow cytometry analysis for cell cycle and apoptosis

Experiments were performed on transfected cells. For cell cycle analysis, cells were treated with 75% ethanol and subsequently stained with propidium iodide (Beyotime, China). The ACEA Novocyte™ flow cytometer (ACEA Biosciences, USA) was utilized for the analysis of the stained cells, resulting in the calculation of the percentage of cells in various phases of the cell cycle (G0/G1, G2/M, and S). The Annexin V-APC/7-AAD Apoptosis Detection Kit (KeyGen Biotech, China) was used to assess cell apoptotic cells by determining the apoptosis rate of the transfected cells according to the manufacturer's guidelines. The ACEA Novocyte™ flow cytometer was then employed to analyze the results.

### Nude mice xenograft experiments

Male BALB/c mice were obtained from Hunan Slake Jingda Experimental Animal Co. Ltd. (Hunan, China) and raised in a laboratory under specific pathogen free-grade conditions. Six mice were kept in cages with a 12-h/12-h light/dark cycle and provided free access to food and water. All animal experiments were performed according to the regulations of the Animal Ethics Committee of Fujian Medical University (IACUC FJMU 2024-Y-0043). The mice were randomly assigned to shC2orf27A-1 and shNC groups (each group comprised six mice). The two groups of nude mice received subcutaneous injections of GC cells stably transfected with shC2orf27A-1 or shNC (0.1mL cell suspension in PBS, 5×10^6^ cells/mL) in the flank. Tumor measurements were taken every 5 days after visible tumors were detected. The tumor volume was determined by applying the formula: tumor volume = (length × width^2^) / 2. Length and width of the tumors were measured using a Vernier caliper in the x/y plane. After 28 days of injection, the mice were euthanized, and the tumors were surgically excised, photographed, weighed, and fixed for immunohistochemistry (IHC) staining and terminal deoxynucleotidyl transferase dUTP nick-end labeling (TUNEL) assay.

### IHC staining and TUNEL assay

Xenograft tumor tissues were first fixed in 4% paraformaldehyde, embedded in paraffin, and sectioned. For IHC staining, the tissue sections underwent dewaxing, dehydration, and rehydration. Citrate buffer was used to retrieve the antigen, and 3.0% hydrogen peroxide was employed to block the endogenous peroxidase activity. After applying a sealing liquid (Beyotime, China), primary antibodies (PCNA at a dilution of 1:600 and MDM2 at a dilution of 1:200; Proteintech) were applied to the sections and incubate overnight at 4°C. The following day, the portions tissues were exposed to HRP-labeled secondary antibodies (Beyotime) at room temperature for 20 min. After washing, streptavidin labeled with HRP was applied and left for 20 min. Subsequently, the DAB chromogen was applied for visualization, followed by hematoxylin counterstain. Apoptosis was assessed using the TUNEL Apoptosis Assay Kit (Beyotime). Tissues were labeled with DAPI to identify nuclei, and apoptotic cells were identified by co-localization of the TUNEL label and DAPI. Micrographs were captured using a fluorescence microscope (NE900, Nexcope, USA).

### Statistical analyses

Data analysis was conducted using R (version 3.6.3), and visualization was performed using either the ggplot2 or Survminer packages. Results from a minimum of three experiments are presented as mean ± SD. The Student's t-test or Wilcoxon rank-sum test was used to compare two groups. Multiple groups were compared using one-way ANOVA, followed by the Tukey's honest significant difference post-hoc test for pairwise comparisons. Survival rates were calculated using the Kaplan-Meier method and compared using log-rank analysis. Correlations among C2orf27A, miR-610, and NOX4 were determined using the Pearson's correlation coefficient. P-values less than 0.05 indicated significant differences.

## Results

### High levels of C2orf27A expression are linked to unfavorable clinical outcomes in GC patients

**Figure 1:**
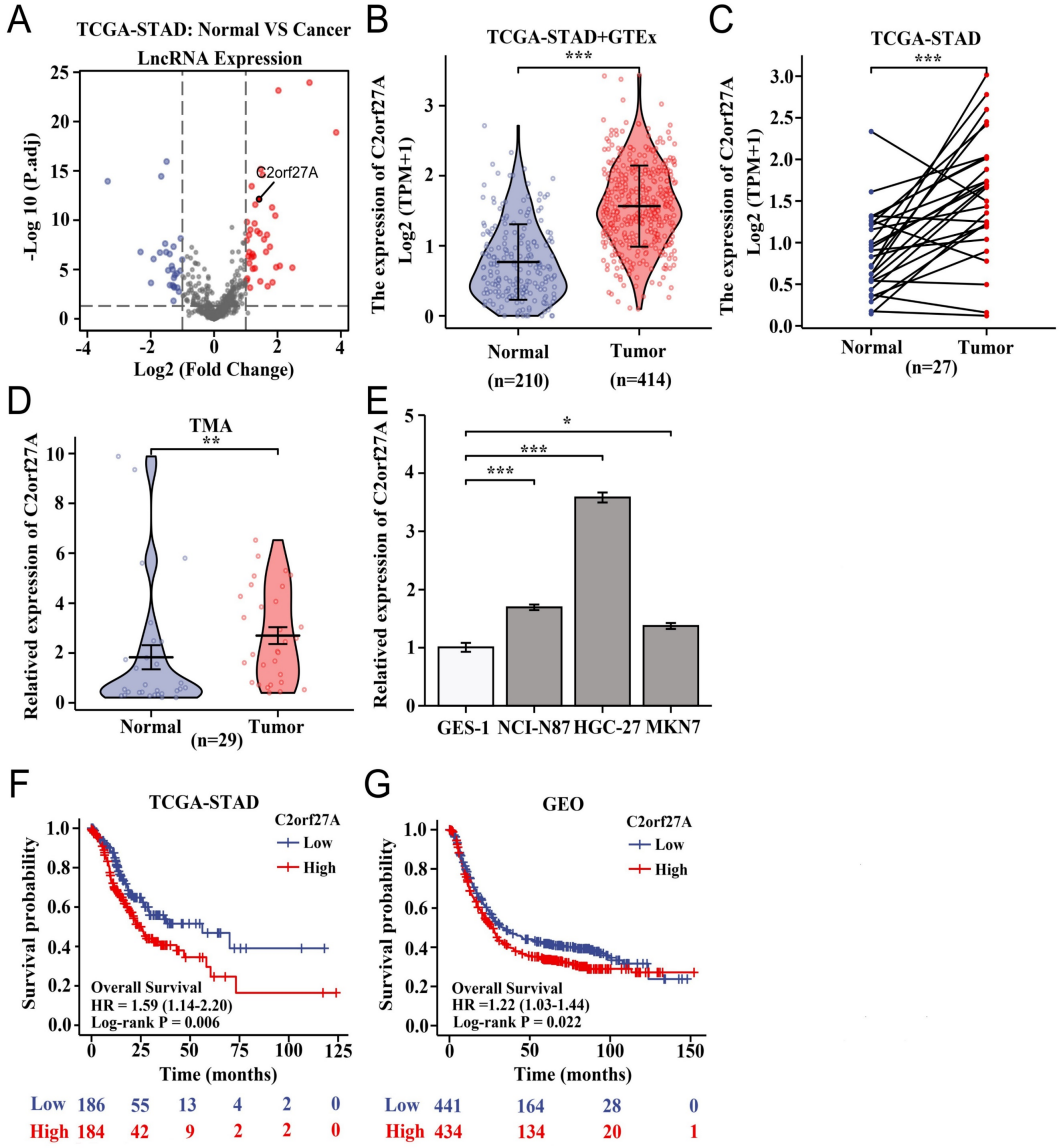
LncRNA C2orf27A is overexpressed and associated with poor clinical outcome in gastric cancer (GC) patients. **(A)** Volcano plot of differentially expressed lncRNAs in GC tissues compared with normal gastric tissues based on TCGA-STAD database (|Log2FC| > 1, P. adj < 0.05). **(B, C)** Expression of C2orf27A in GC tissues compared with that in normal gastric tissues using unpaired and paired samples from TCGA-STAD and GTEx database. **(D)** Expressions of C2orf27A in GC and normal gastric tissues from a commercially available GC tissue microarray (TMA) examined by qRT-PCR. **(E)** Relative expression of C2orf27A in GC and normal gastric epithelial cell lines. **(F, G)** Overall survival rates of GC patients with low and high C2orf27A levels analyzed using the Kaplan-Meier method. Patient data from TCGA-STAD and six GEO datasets were assigned into two subgroups according to the median expression of C2orf27A. *P < 0.05, **P < 0.01, and ***P <0.001.

We analyzed lncRNA-seq data for C2orf27A from three public databases (TCGA-STAD, GTEx, and GEO) using bioinformatic techniques. In our study, the expression of C2orf27A was notably elevated in GC tissues, that in normal gastric tissues (Fig. 1A-C). To confirm our results, we performed qRT-PCR for the expression of C2orf27A in GC tissues and adjacent normal tissues from a commercially available TMA containing 29 patients. These findings are consistent with those of our bioinformatics analysis (Fig. 1D). In three GC cell lines (NCI-N87, HGC-27and MKN-7), we observed a significant increase in the expression of C2orf27A compared to that in the normal human gastric epithelial cell line (GES-1) (Fig. 1E). In addition, we used TCGA-STAD and six GEO datasets to evaluate the prognostic importance of C2orf27A expression. Compared to that in patients with low C2orf27A expression, high C2orf27A expression in GC patients was associated with decreased overall survival rates, as shown in the Kaplan-Meier survival curves (Fig. 1F, G). These results suggest that the expression of C2orf27A could be used as an indicator of unfavorable outcomes in patients with GC.

### C2orf27A knockdown suppresses GC cell proliferation and enhances their apoptosis *in vitro*

**Figure 2:**
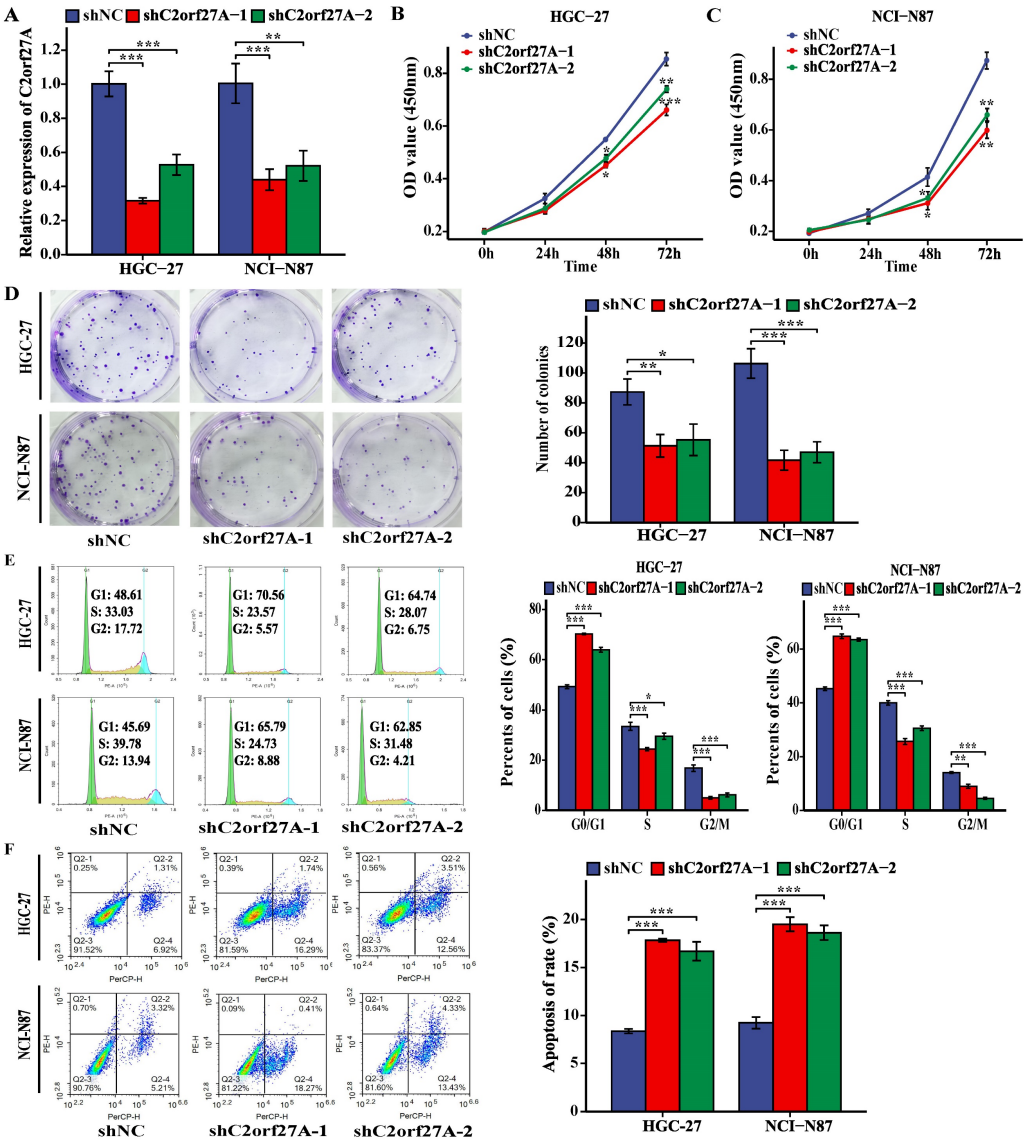
Knockdown of C2orf27A inhibits proliferation and promotes death of GC cells *in vitro*. **(A)** The knockdown efficiency of C2orf27A was detected in HGC-27 and NCI-N87 cells using qRT-PCR. **(B, C, D)** CCK-8 and colony formation assays were used to assess the proliferation of HGC-27 and NCI-N87 cells after knockdown of C2orf27A. **(E, F)** Flow cytometry was applied to evaluate the cell cycle and apoptosis of HGC-27 and NCI-N87 cells after knockdown of C2orf27A. Data are presented as the mean ± SD of three independent experiments. *P < 0.05, **P < 0.01, and ***P <0.001.

Based on the high C2orf27A expression in the HGC-27 and NCI-N87 cell lines (Fig. 1E), they were selected for further experiments. shRNAs targeting C2orf27A (shC2orf27A-1 and shC2orf27A-2) were used to transfect HGC-27 and NCI-N87 cells. To confirm the knockdown efficiency, qRT-PCR analysis was performed (Fig. 2A). C2orf27A knockdown significantly reduced the proliferative capacity of both GC cell lines, as shown by the results of the CCK-8 and colony formation experiments (Fig. 2B-D). Flow cytometry showed that the number of GC cells in the G0/G1 phase increased significantly after C2orf27A knockdown, with a decrease in the number of GC cells in the S and G2/M phase, when compared to that of the control group (Fig. 2E). This finding suggests that downregulated C2orf27A expression blocked the entry of GC cells into the S and G2/M phases. Conversely, flow cytometry indicated that reducing C2orf27A expression positively impacted GC cell apoptosis (Fig. 2F). In summary, blocking C2orf27A resulted in the reduced growth and increased death of GC cells in a laboratory setting.

### C2orf27A knockdown inhibits GC cell proliferation and facilitates their apoptosis *in vivo*

**Figure 3:**
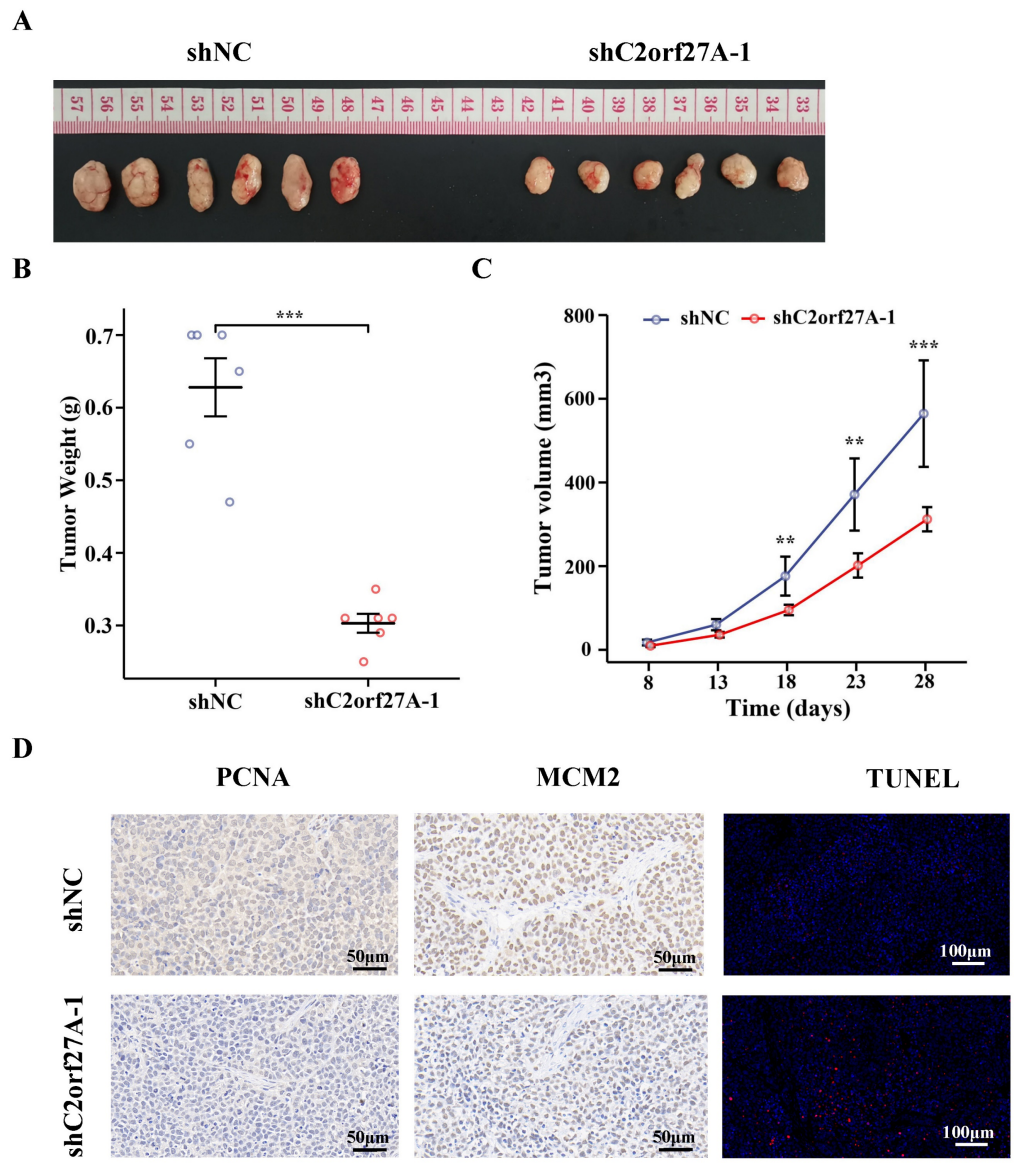
Knockdown of C2orf27A suppresses proliferation and facilitates death of GC cells *in vivo*. **(A)** Representative images of subcutaneous xenograft tumors after injection of C2orf27A knockdown or negative control GC cells. **(B)** Weights of subcutaneous tumors were measured on day 28. **(C)** Volumes were evaluated every 5 days after the formation of subcutaneous tumors. **(D)** Representative images of immunohistochemistry staining with antibodies against PCNA and MCM2 in subcutaneous xenograft specimens (40x). TUNEL apoptosis assay showing the apoptotic cells in subcutaneous tumors (20x). **P < 0.01, and ***P <0.001.

To further verify the role of C2orf27A in GC, we subcutaneously injected HGC-27 cells transduced with shC2orf27A-1 or shNC into nude mice to generate xenograft model. The results revealed substantial impairment in tumor growth upon shC2orf27A knockdown (Fig. 3A). Tumors with C2orf27A knockdown had lower weights and smaller volumes than those of control tumors (Fig. 3B, C). IHC staining revealed a noticeable suppression of PCNA and MCM2 expression upon C2orf27A knockdown, whereas TUNEL-positive cells exhibited the opposite effect (Fig. 3D).

Collectively, these findings suggest that C2orf27A suppresses the growth of GC cells and promotes their programmed cell death in living organisms, which is consistent with findings from experiments conducted *in vitro*.

### C2orf27A decreases miR-610 expression

**Figure 4:**
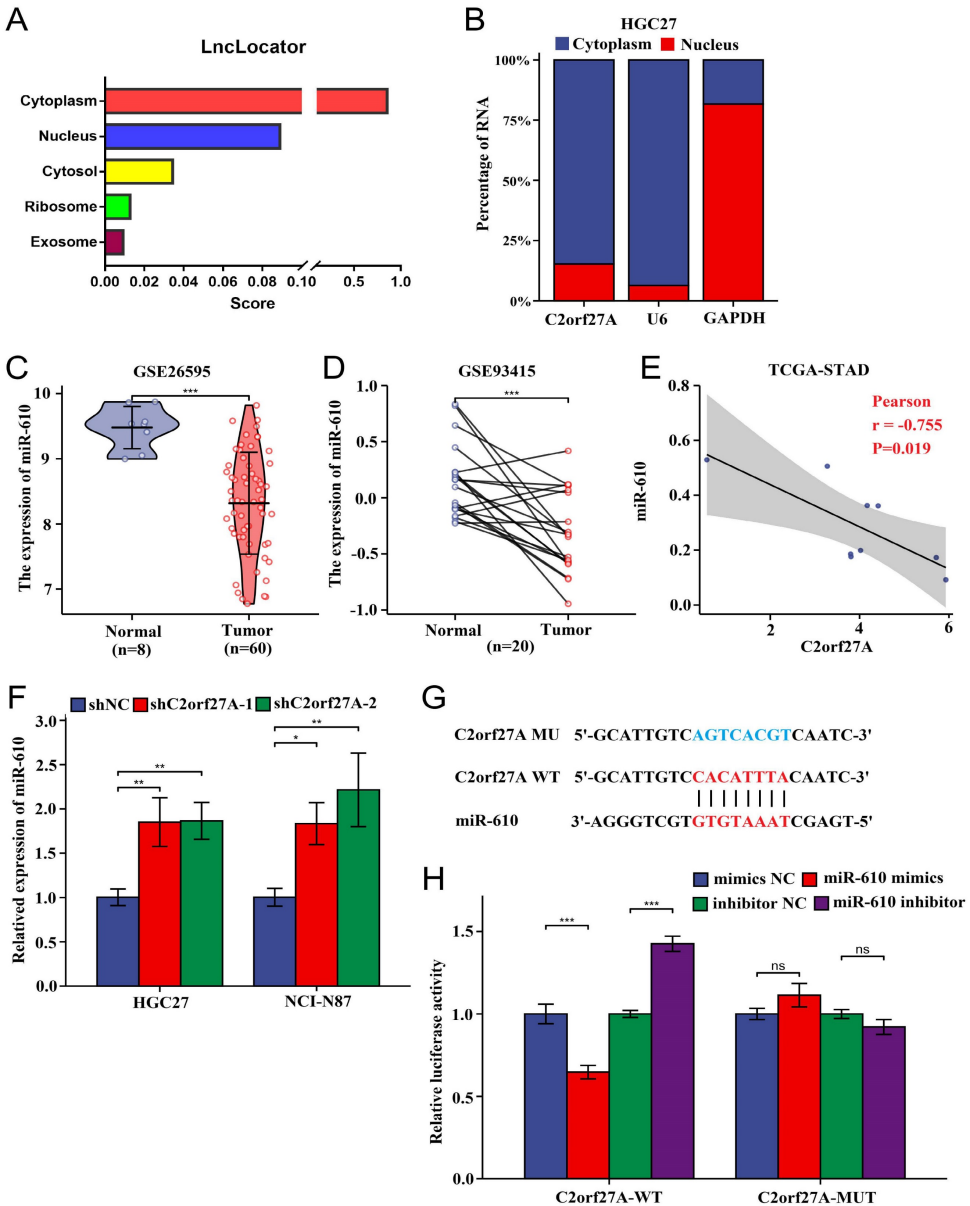
C2orf27A functions as a molecular sponge of miR-610 in GC cells.** (A)** The cellular localization of C2orf27A was predicted using lncLocator. **(B)** A nucleocytoplasmic separation experiment was used to detect the subcellular localization of C2orf27A in HGC-27 cells. **(C, D)** GSE26595 and GSE93415 datasets from GEO indicate that miR-610 expression was markedly lower in GC than in normal gastric tissues (unpaired and paired samples). **(E)** Correlation analysis of C2orf27A and miR-610 expression in GC based on TCGA-STAD. **(F)** qRT-qPCR was used to detect miR-610 levels after C2orf27A knockdown in HGC-27 and NCI-N87 cells. Data are presented as the mean ± SD of three independent experiments. **(G)** Bioinformatics analysis using LncTar showed the putative binding site between C2orf27A and miR-610. **(H)** Luciferase reporter gene assays showed that miR-610 negatively regulated the luciferase activity of C2orf27A-WT, rather than that of the mutant C2orf27A-MUT. *P < 0.05, **P < 0.01, and ***P <0.001, ns: no significance.

LncRNAs have been found to function as ceRNAs that specifically bind to miRNAs in the cytoplasm 14. Using the lncLocator database, we predicted that intracellular C2orf27A is predominantly localized in the cytoplasm (Fig. 4A). Subcellular fractionation assays of HGC-27 cells confirmed this prediction (Fig. 4B). Previous studies have reported that miR-610 acts as a tumor suppressor 8, 11. Based on the information provided for GC from GEO, miR-610 levels were considerably lower than those of normal gastric tissues (Fig. 4C, D). Furthermore, an inverse relationship was observed between C2orf27A and miR-610 levels in TCGA-STAD database (Fig. 4E). We further explored whether C2orf27A directly binds to miR-610. As revealed by the online predictive tool (LncTar), a putative binding site existed between C2orf27A and the miR-610 sequence, indicating miR-610 may be ceRNAs of C2orf27A (Fig. 4F). We used a double-luciferase reporter assay to test this hypothesis. HEK-293 cells were transfected with the C2orf27A-WT, a mutated C2orf27A reporter vector (C2orf27A -MUT), miR-610 mimics, and a miR-610 inhibitor simultaneously. Compared to that in the control group, the transfection of miR-610 mimics attenuated the luciferase activity of the C2orf27A-WT group without affecting the C2orf27A-MUT group. By contrast, the miR-610 inhibitor significantly enhanced the luciferase activity in the C2orf27A-WT group, but not in the C2orf27A-MUT group (Fig. 4G). Additionally, the inhibition of C2orf27A significantly increased the levels of miR-610 in both HGC-27 and NCI-N87 cells (Fig. 4H). These data suggest that C2orf27A functions as a ceRNA to efficiently suppress miR-610 expression.

### Inhibition of miR-610 reverses the effect of C2orf27A knockdown on the aggressive behavior of GC cells

**Figure 5:**
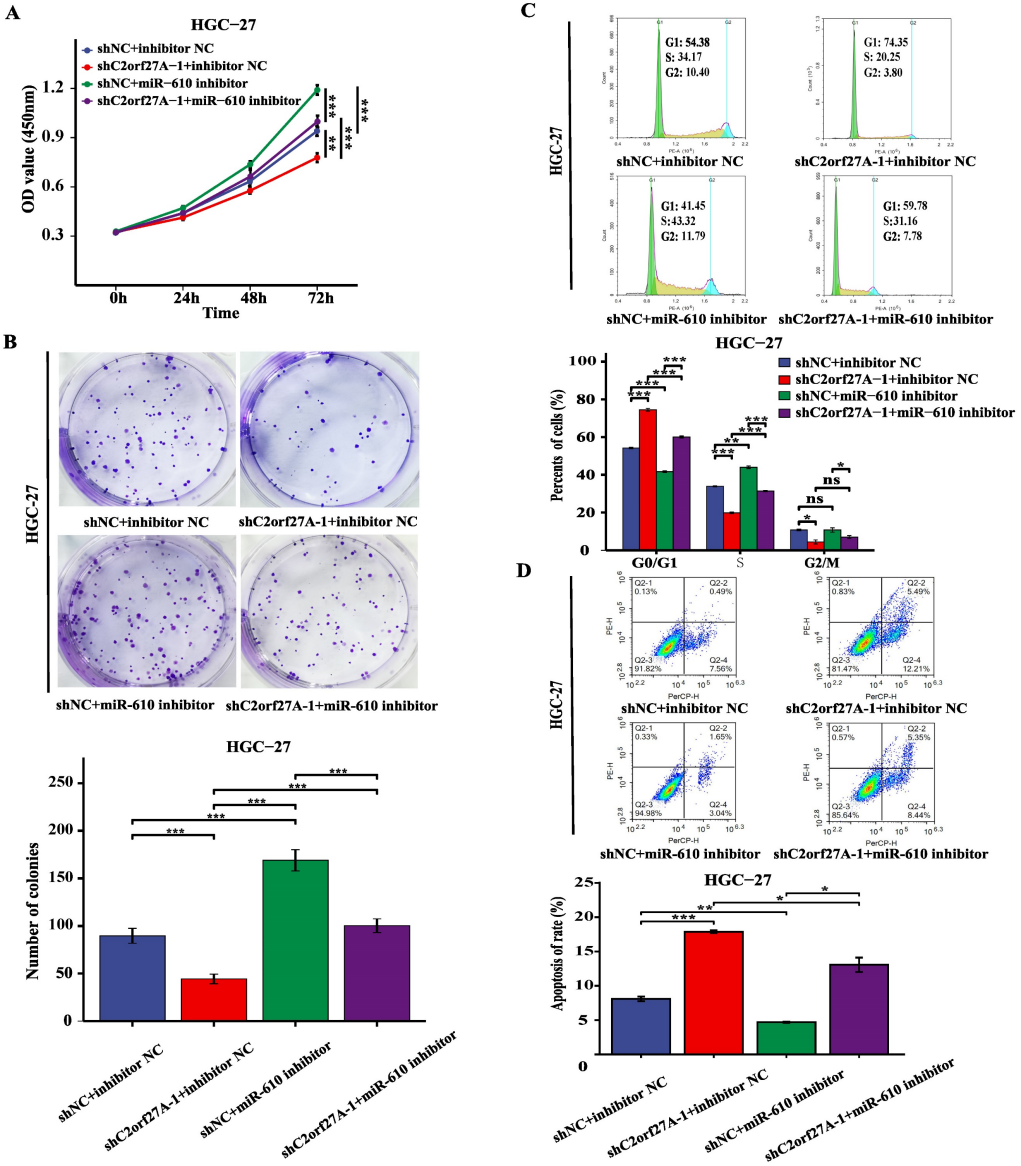
MiR-610 inhibition partially counteracts the antitumor effects of C2orf27A knockdown in GC cells. MiR-610 inhibitor partially reversed the effects of C2orf27A knockdown on the proliferation **(A)**, colony formation **(B)**, cell cycle progression **(C)** and apoptosis **(D)** of HGC-27 cells. Data are presented as the mean ± SD of three independent experiments. *P < 0.05, **P < 0.01, and ***P <0.001, ns: no significance.

A rescue experiment was conducted in HGC-27 cells by knocking down C2orf27A and transfecting them with miR-610 inhibitors to investigate the potential impact of C2orf27A on GC progression through miR-610. The CCK8, colony formation and cell cycle assay showed that the miR-610 inhibitor partially abrogated the suppression of proliferation caused by C2orf27A knockdown in HGC-27 cells (Fig. 5A-C). By contrast, the miR-610 inhibitor attenuated the enhanced apoptosis induced by the C2orf27A knockdown in HGC-27 cells (Fig. 5D). These findings indicate that C2orf27A controls the growth and death of GC cells through miR-610.

### MiR-610 inhibits the proliferation and enhances death of GC cells by directly targeting NOX4

**Figure 6:**
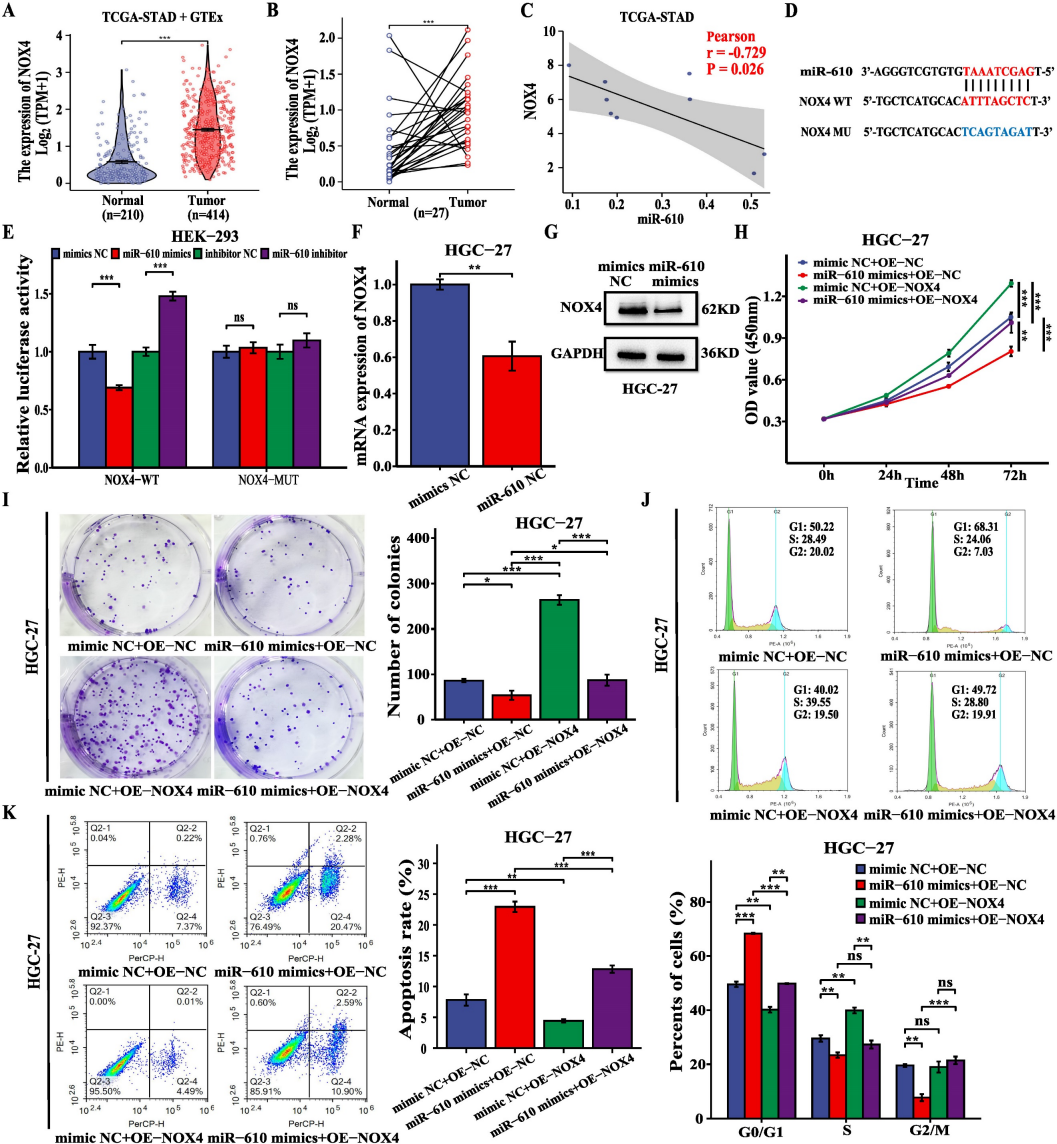
MiR-610 blocks the malignant behavior of GC cells by inhibiting NOX4 expression. **(A, B)** The data from TCGA-STAD and GTEx indicated that NOX4 expression was significantly higher in GC tissues than that in normal gastric tissues (unpaired and paired samples). **(C)** The correlation analysis of miR-610 and NOX4 expression in GC tissues based on TCGA-STAD. **(D)** Bioinformatics analysis using DIANA-microT-CDS showed the putative binding site between miR-610 and NOX4. **(E)** Luciferase reporter gene assays revealed that miR-610 negatively regulated the luciferase activity of NOX4-WT rather than that of the mutant NOX4-MUT. **(F, G)** qRT-PCR and western blot were used to detect NOX4 levels after transfecting miR-610 mimics into HGC-27 cells. **(H-K)** NOX4 overexpression partially offset the functions of miR-610 mimics on proliferation, colony formation, cell cycle progression, and apoptosis of HGC-27 cells. Data are presented as the mean ± SD of three independent experiments. *P < 0.05, **P < 0.01, and ***P <0.001, ns: no significance.

Previous studies have reported that NOX4 plays a key oncogenic role in GC (12). Analysis of TCGA-STAD and GTEx data revealed a notable increase in NOX4 expression in GC tissues compared to that in normal samples (Fig. 6A, B). In addition, miR-610 was negatively correlated with NOX4 based on TCGA-STAD database (Fig. 6C). We investigated whether miR-610 directly binds to NOX4. From the predicted data in the online database (DIANA-microT-CDS), a putative binding region was identified between NOX4 and miR-610 (Fig. 6D). Dual-luciferase reporter assay was performed on HEK-293 cells to validate the mechanistic connection between miR-610 and NOX4. HEK-293 cells were transfected with NOX4-WT, a mutated NOX4 reporter vector (NOX4-MUT), miR-610 mimics, and a miR-610 inhibitor simultaneously. Compared to that in the control group, miR-610 mimics attenuated the luciferase activity in the NOX4-WT group without affecting the NOX4-MUT group. However, the miR-610 inhibitor significantly enhanced luciferase activity in the NOX4-WT group but not in the NOX4-MUT group (Fig. 6E). In addition, the miR-610 mimics significantly suppressed NOX4 mRNA and protein expression in HGC-27 cells (Fig. 6F, G). Additionally, we conducted a rescue experiment by increasing the expression of NOX4 and introducing miR-610 mimics into HGC-27 cells. In HGC-27 cells, CCK-8 assay, colony formation assay, and cell cycle analysis indicated that NOX4 overexpression reduced the inhibitory impact on cell growth from miR-610 mimics (Fig. 6H-J). Conversely, upregulation of NOX4 mitigated the increase in apoptosis induced by miR-610 mimics in HGC-27 cells (Fig.6K). These findings indicate that miR-610 regulates the growth and death of GC cells by targeting NOX4.

### C2orf27A regulates NOX4 expression to promote oncogenic activities in GC cells

**Figure 7:**
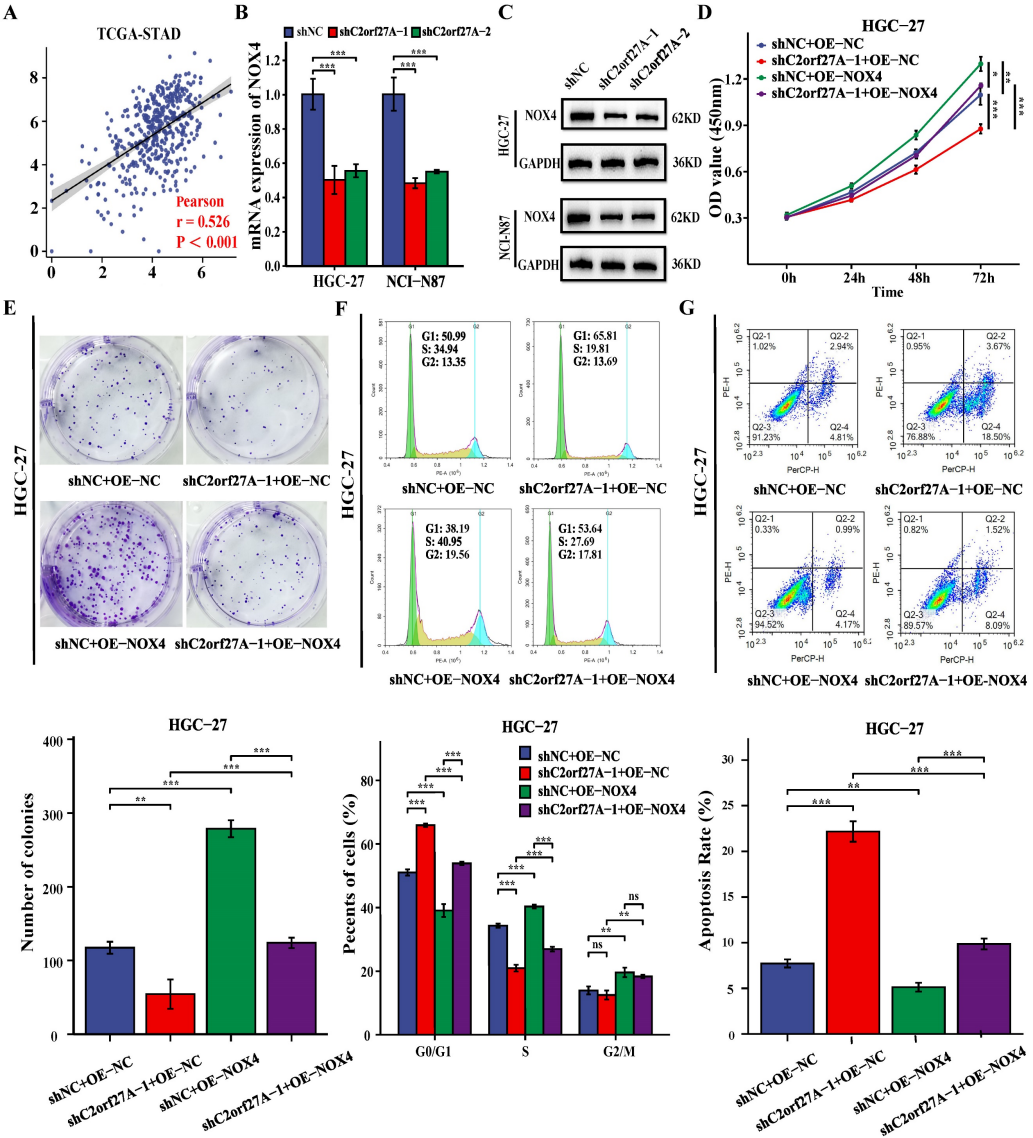
C2orf27A knockdown exerts antitumor effects in GC cells by downregulating NOX4 expression. **(A)** Correlation analysis of C2orf27A and NOX4 expression in GC cells based on TCGA-STAD. **(B, C)** qRT-PCR and western blot detected the levels of NOX4 in HGC-27 and NCI-C87 cells after C2orf27A knockdown. **(D-G)** NOX4 overexpression partially offset the effects of C2orf27A knockdown on proliferation, colony formation, cell cycle progression and apoptosis of HGC-27 cells. Data are presented as the mean ± SD of three independent experiments. *P < 0.05, and **P < 0.01, ***P <0.001, ns: no significance.

A positive correlation between C2orf27A and NOX4 levels was obtained by analyzing TCGA-STAD database (Fig. 7A). To investigate the regulation of NOX4 expression in GC cells by C2orf27A, we transfected HGC-27 and NCI-N87 cells with shC2orf27A-1 or shC2orf27A2. RT-qPCR and western blot analyses revealed a significant decrease in NOX4 expression at both the mRNA and protein levels, respectively, following C2orf27A knockdown (Fig. 7B, C). We designed rescue experiments to assess the effect of NOX4 on the pro-tumorigenic role of C2orf27A in GC. The inhibitory effect of C2orf27A knockdown on cell proliferation was partially counteracted by the overexpression of NOX4 (Fig. 7D-F). Conversely, the inhibitory effect of NOX4 overexpression on apoptosis counteracted the promoting effect of C2orf27A knockdown (Fig. 7G). Overall, these results revealed that C2orf27A influences the growth and death of GC cells by promoting NOX4.

## Discussion

GC, a prevalent type of digestive system tumor placing a growing strain on healthcare worldwide. Hence, exploring new early indicators and treatment objectives is crucial for enhancing the clinical approaches and outcomes of patients with GC. Increasing evidence suggests that lncRNAs are disrupted in various human cancers and participate in cancer development and progression. Dysregulated lncRNAs may act as oncogenes or tumor suppressors and influence cancer development 15-17. A previous study using bioinformatics prediction reported that C2orf27A might be one of the key upstream factors regulating hub ferroptosis-related genes in GC 6. In this study, we assessed the importance and role of C2orf27A in GC. Our findings indicate a notable increase in C2orf27A levels in both GC tissues and cell lines. High C2orf27A levels are linked to decreased overall survival in patients with GC. Additional functional tests showed that the suppression of C2orf27A significantly reduced GC cell growth and increased their apoptosis both *in vitro* and *in vivo*. These results are consistent with previous report that implicate C2orf27A as an oncogene in HCC 5.

Multiple studies have shown that cytoplasmic lncRNAs can act as ceRNAs by competing for miRNA binding, leading to the regulation of target mRNA expression by sharing MREs. Numerous ceRNA networks are dysregulated during tumor development and progression 18, 19. To identify potential miRNAs associated with C2orf27A, we merged the miRNA and mRNA expression matrices from the TCGA-STAD dataset. By selecting the expression data of C2orf27A, we performed a correlation analysis using the Pearson correlation test. This exhaustive process identified 2028 miRNAs exhibiting varying degrees of correlation with C2orf27A expression. Notably, 11 of these miRNAs showed significant negative correlations (r < -0.70, p < 0.05, data not shown), indicating a potentially important regulatory relationship. Among these, we selected miR-610 due to its well-documented role as a tumor suppressor in diverse cancer types, including GC 9, 20-22. MiR-610 levels are lower in GC tissues than in normal adjacent tissues. Moreover, initiation of the EGF signaling pathway led to an increase in VASP expression and a decrease in miR-610 expression. MiR-610 binds directly to the 3' untranslated region of VASP mRNA, preventing its translation and inhibiting VASP-induced GC cell migration and invasion 11. Zeng and colleagues discovered that decreased levels of miR-610 led to the activation of the Wnt/β-catenin pathway by inhibiting LRP6 and TBL1X, ultimately advancing the development of HCC 8. Moreover, miR-610 is a direct target of multiple lncRNAs and circRNAs 23-28. Our current research showed a notable decrease in miR-610 expression, which was linked to C2orf27A expression in GC, through analysis of TCGA-STAD and GEO databases. Additionally, C2orf27A was found to directly interact with and suppress miR-610 expression, as determined by bioinformatics analyses and luciferase reporter assays. Furthermore, C2orf27A knockdown inhibited the proliferation and promoted the apoptosis of GC cells, which was partially reversed by synergistic knockdown of miR-610. Consequently, our findings corroborate the notion that C2orf27A possesses oncogenic properties, partly, by functioning as a miR-610 sponge in GC cells.

To identify potential mRNAs associated with C2orf27A, we conducted a comprehensive analysis using the Pearson correlation test to examine the relationship between the expression levels of all 20,503 genes and that of C2orf27A within the TCGA-STAD dataset. Notably, eight coding genes emerged as significantly positively correlated with C2orf27A (r > 0.50, p < 0.05, data not shown). Among this select group of genes, we deliberately chose NOX4 for further investigation due to its well-documented role as a tumor promoter in gastric cancer. Previous studies have indicated that NOX4 expression is increased in GC and is associated with larger tumor size, lymphatic spread, blood vessels invasion, and unfavorable outcomes in patients with GC 12, 29. Furthermore, knockdown of NOX4 suppresses the proliferation and invasion of GC cells, while promoting and sensitizing them to apoptosis and anoikis *in vitro* 13, 30, 31. Through our rigorous examination of the TCGA-STAD dataset, we reinforced the finding that NOX4 displays augmented expression in GC tissues in comparison to adjacent normal tissues. Strikingly, this elevated NOX4 expression exhibits an inverse relationship with the level of miR-610. Utilizing advanced online predictive software, we uncovered potential interactive sites between miR-610 and NOX4 sequences. This was further verified experimentally by dual-luciferase reporter assays, including mutagenesis tests. Further, our q-PCR and Western Blot analyses underscored the significant reduction in NOX4 mRNA and protein levels upon miR-610 overexpression in GC cells. Intriguingly, functional rescue experiments illuminated an intricate interplay, as the concurrent overexpression of NOX4 during miR-610 mimic transfection partially mitigated the miR-610-mediated suppression of GC cell proliferation and potentiation of apoptosis. Additionally, we observed that the suppression of C2orf27A effectively repressed NOX4 expression at both the transcriptional and translational levels, with functional rescue experiments revealing that the concurrent upregulation of NOX4 upon C2orf27A knockdown could partially reverse the C2orf27A-induced anti-proliferative and pro-apoptotic effects on GC cells.

In summary, our findings elucidate a novel mechanism where cytoplasmic C2orf27A acts as a sponge for miR-610, thereby de-repressing NOX4, which in turn fosters GC cell proliferation and inhibits apoptosis. This not only sheds light on a fundamental biological process but also suggests a promising avenue for advancing novel diagnostic and therapeutic modalities in the treatment of GC patients. The elevated expression of C2orf27A observed in GC tissues, coupled with its correlation to unfavorable patient outcomes, underscores its potential as a valuable biomarker for early detection and risk assessment of GC. Furthermore, as C2orf27A functions as a competitive endogenous RNA by sequestering miR-610, which subsequently leads to the upregulation of NOX4, targeted interventions aimed at inhibiting C2orf27A represent a novel therapeutic strategy that holds the potential to disrupt this oncogenic pathway, offering a novel treatment option for GC. However, there are several limitations and challenges that need to be addressed before translating these discoveries into clinical application. Crucially, larger-scale, multi-center studies are indispensable to substantiate the prognostic worth of C2orf27A in GC and meticulously evaluate its biomarker potential. Furthermore, extensive research endeavors are imperative to unravel the intricate regulatory network encompassing C2orf27A, miR-610, and NOX4, while also uncovering additional downstream targets that may play pivotal roles in GC progression. Only through such meticulous and comprehensive investigations can we harness the full potential of these molecules for clinical benefit.

## Supplementary Material

Supplementary tables.
