# Supplementary material for: LncRNA C2orf27A Promotes Gastric Cancer by Sponging MiR-610 and Elevating NOX4 Expression
Source: J Cancer. 2025 Jan 27;16(5):1504–18. doi: 10.7150/jca.100621 (PMC11843226; doi:10.7150/jca.100621)
Supplement: Supplementary file 1 — Supplementary tables. [file jcav16p1504s1.pdf]

Table S1. The sequences of the shRNAs and oligonucleotides

Name	Sequence (5'−3')
shC2orf27A-1	AGCATTGTCCACATTTACAAT
shC2orf27A-2	ATCAGCATTGTCCACATTTAC
miR-610 mimics	UGAGCUAAAUGUGUGCUGGGACCAGCACACAUUUAGCUCAUU
miR-610 inhibitor	UCCCAGCACACAUUUAGCUCAUU
mimic NC	UCACAACCUCCUAGAAAGAGUAGAUCUACUCUUUCUAGGAGGUUGUGA
inhibitor NC	UCUACUCUUUCUAGGAGGUUGUGA

Table S2. Primers for qRT-PCR

Name	Forward primer (5'−3')	Reverse primer (5'−3')
C2orf27A	CAACAGTATGGCTCCAAATGATG	GAAGGGATAAAGTAACTGTGGTGG
miR-610	GCCGTGAGCTAAATGTGTG	CAGTGCGTGTCGTGGA
NOX4	TGACGTTGCATGTTTCAGGAG	AGCTGGTTCGGTTAAGACTGAT
U6	CTCGCTTCGGCAGCACA	AACGCTTCACGAATTTGCGT
GAPDH	ACAGCCTCAAGATCATCAGC	GGTCATGAGTCCTTCCACGAT
